# An Up-Scalable and Cost-Effective Methodology for Isolating a Polypeptide Matrix Metalloproteinase-9 Inhibitor from *Lupinus albus* Seeds

**DOI:** 10.3390/foods10071663

**Published:** 2021-07-19

**Authors:** Joana Mota, Maria E. Figueira, Ricardo B. Ferreira, Ana Lima

**Affiliations:** 1 LEAF, Instituto Superior de Agronomia, Universidade de Lisboa, 1349-017 Lisbon, Portugal; rbferreira@isa.ulisboa.pt (R.B.F.); agusmaolima@gmail.com (A.L.); 2 Research Institute for Medicines and Pharmaceutical Sciences (iMed.UL), Faculty of Pharmacy, University of Lisbon, Av. Prof. Gama Pinto, 1649-003 Lisboa, Portugal; efigueira@ff.ulisboa.pt; 3 Faculty of Veterinary Medicine, Universidade Lusófona de Humanidades e Tecnologias, Campo Grande, 376, 1749-024 Lisbon, Portugal

**Keywords:** MMP-9, HT29, GRAS-safe, deflamin, white lupin, nutraceutical

## Abstract

One of the most challenging problems with food-borne bioactive compounds is that there are commonly no cost-effective, generally recognized as safe (GRAS) methods for obtaining gram quantities of their purified forms. Here we aimed at developing a method to isolate deflamin, an oligomeric protein from lupin seeds with anti-inflammatory and anticancer activity through matrix metalloprotease (MMP)-9 inhibition. Our goal was to develop a GRAS method that could be easily up-scalable whilst maintaining deflamin’s activity. A sequential precipitation methodology was developed, using an aqueous extraction, followed by heat denaturation, acid precipitation and solubilization in ethanol. A final precipitation with 90% ethanol yielded a purified protein which was sequenced through mass spectrometry and tested for its MMP inhibitory activity using the Dye-quenched (DQ) gelatin assay and the standard wound healing assay in HT29 cells. The developed method yielded a purified oligomer, which represented 0.1% (*w*/*w*) of total dry seed weight and was positively confirmed to be deflamin. It further showed to effectively reduce MMP-9 gelatinolytic activity as well as colon cancer cell migration, hence corroborating the effectiveness of our method. Overall, this is the first reported method for isolating an MMP-9 inhibitor from legume seeds, which is up-scalable to an industrial level, in a cost-effective manner.

## 1. Introduction

Nutraceuticals and functional foods have become an important focus for food manufacturers and consumers [1]. As nutritional alternatives for disease management and health promotion become more available in the market, both in conventional foods as well as individual supplements, novel candidates with high applicability and easiness to manufacture industrially are being pursued, particularly in the case of cancer [1]. Since rapid metastization is the major contributor to cancer mortality [2], its mediators have become one of the most important therapeutic goals, particularly a group of matrix metalloproteinases (MMPs) named MMP-2 and MMP-9, which are important regulators to the key processes underlying metastasis, including cell adhesion, spreading, migration, invasion and angiogenesis, and pre-cancer inflammatory diseases such as colitis [3,4]. There is a considerable increasing larger body of evidence with pre-clinical and clinical tests showing that MMP inhibition reduces cancer growth and metastization, hence making these gelatinases a good therapeutic target to prevent or reduce several types of cancer [4,5]. 

Previous efforts to target MMP-9 and other MMPs used broad- spectrum or semi-selective inhibitors (MMPIs). Well-known examples are provided by tetracycline, zoledronate, ethylenediaminetetraacetic acid (EDTA), 1,10-phenanthroline, 2S,3R-3-amino-2-hydroxy-4-(4-nitrophenyl)butanoyl-L-leucine, and neovastat^®^. Up to now, a considerable number of MMPIs have also been synthesized, some of which have been used as potential therapeutic agents to limit tumor progression [6]. However, only a few of these MMPIs entered the clinical stage, and while they did show signs of some success in patients [7], because of MMP’s ubiquity, most trials were hampered by dose-limiting toxicity, insufficient clinical benefits, and severe side-effects, due to their lack of specificity and inhibition of normal physiological processes. 

One way to surpass these setbacks would be to discover MMPIs capable of acting directly in loco, without affecting MMP intracellular expression and therefore avoiding generalized side-effects [6,7]. This could be of particular interest in the case of gastrointestinal cancers, which are undoubtedly the most diet-linked types of cancer [8]. Indeed, a substantial amount of research has turned towards the discovery of novel plant-food-derived MMPIs. Such is the case of several compounds present in legume seeds [9], which have been reported to exhibit a benefic role as anticancer and antimetastatic agents in various animal models [10]. However, none of these compounds has been effectively isolated for nutraceutical purposes and those which have, such as the case of lunasin, have no apparent effect on MMPs [11,12]. Also, other bioactives in legume seeds such as polyphenols, saponins, and protease inhibitors with anticancer activities [10] may also present some constraints, as the fact that some are considered to be anti-nutrients and/or can exert cytotoxicity at high levels. Findings in our group provided a new alternative on this matter when we discovered a new MMPI oligomeric protein, isolated from the edible seeds of sweet lupin *Lupinus albus* [13,14]. This MMPI (named deflamin, patent WO/2018/060528) is one of the few proteins MMPIs derived from staple foods that is nontoxic and effectively reduces MMP-2 and -9 activities in vivo [15]. Because it acts in situ and, apparently, is not absorbed this deflamin bypasses most problems related to MMPIs in clinical trials, associated to high toxicity and systemic secondary effects [15]. Being a food component, with a potentially high anti-tumor function, deflamin could be used as a nutraceutical, or a functional food or as a nutritional complement for colorectal cancer (CRC) patients. Nonetheless, when considering its incorporation in diets, deflamin is present in very low amounts in the seeds [15], hence a diet on lupin seeds *per se* would not suffice to exert the necessary effects. In fact, this is the one of the most challenging problems with foodborne anticancer compounds [16]. They often never reach clinical or even pre-clinical trials because their production is hampered by the cost of their synthetic production, or there are no cost-effective methods for obtaining gram quantities of their highly purified forms [17]. In the case of deflamin, it may be possible to surpass this by using some of its unique properties, such as resistance to boiling and high-water solubility. This could be particularly important, as the development of a specific methodology that could concentrate the bioactive protein in lupin, to introduce it in physiological active quantities in food systems could propose a simple approach to develop innovative functional foods that may have physiological benefits or decrease the risks of disorders. Indeed, it has been referred that food proteins exhibit excellent potential for developing and engineering a variety of new GRAS matrices with the potential to incorporate nutraceutical compounds and give controlled release via the oral administration [18]. Furthermore, food protein concentrates present clear advantages, including their high nutritional value, abundant sources, and acceptability as naturally occurring food elements [19]. So, under this context, we set out to develop a method to concentrate and isolate deflamin from lupin seeds, which could be easily up-scalable and used to introduce it in foods. Overall, the developed method could yield the perfect delivery for deflamin, as a GRAS-safe food component or as an isolated nutraceutical, to be used in preventive/curative approaches to gastrointestinal diseases.

## 2. Materials and Methods

### 2.1. Protein Extraction Isolation

Dry, mature seeds of *Lupinus albus* L. (lupin) were used in this work. The MMPI protein extract was isolated taking advantages of its several features: high water solubility, low molecular mass, ability to resist boiling and acid denaturation, and by conjugation of previously described methods [14,20], with several modifications. Briefly, approximately 100 g ± 0.1 g of dry lupin seed was extracted using 50 mM of Tris-HCl buffer, pH 7.5 (1:10, *w*/*v*). The homogenate was centrifugated at 13,500× *g* for 30 min at 4 °C yielding the buffer extract (BE). The supernatant was collected, boiled for 10 min and centrifugated at 13,500× *g* for 20 min at 4 °C. Subsequently, the supernatant was made to pH 4.0 and centrifugated at 13,500× *g* for 20 min at 4 °C. The pellet was resuspended in 40% (*v*/*v*) ethanol containing 0.4 M NaCl, and centrifugated at 13,500× *g*, 30 min, 4 °C. The supernatant was made to 90% (*v*/*v*) ethanol and left overnight at −20 °C. The following day, the mixture was centrifugated at 13,500× *g* for 30 min at 4 °C and the pellet was resuspended in the smallest possible volume of milli-Q water. The extract obtained, containing isolated deflamin, was stored frozen in falcon tubes at −80 °C.

### 2.2. Sodium Dodecyl Sulfate-Polyacrylamide Gel Electrophoresis

Samples were treated according to Lima et al. [14]. One-dimensional electrophoresis was carried out, following the method described by Laemmli [21]. Gels were fixed for 1 h in 50% (*v*/*v*) methanol and 2% (*v*/*v*) of ortho-phosphoric acid, washed 3 times with milli-Q water and stained in 0.5% (*w*/*v*) Comassie Brilliant Blue G-250 in 34% (*v*/*v*) methanol, 17% (*w*/*v*) ammonium sulphate, and 2% (*v*/*v*) ortho-phosphoric acid for 2 h. Destaining was performed with milli-Q water until polypeptide bands were clearly visible against a clear background.

### 2.3. Reverse Gelatin Zymography

Reverse zymography, used to detect and quantify potential proteins against MMP-9 activity in different samples, was performed as described by Lima et al. [14]. A white background against dark bands marked the MMPI inhibition of gelatin degradation.

### 2.4. Mass Spectrometry Analysis 

Selected isolated peaks were analyzed on a 5600 TripleTOF mass spectrometer according to the method described by Trindade et al. [22]

Protein identification was obtained using Protein Pilot™ software (v 5.0, ABSciex^®^) with the subsequent search parameters: identification from uniprot database, with no alkylation or digestion for the peptide samples. As a criteria for protein filtering, we used 1.3 unused score value and a 95% peptide confidence filtering and >0 contribution.

### 2.5. MMP-9 and MMP-2 Catalytic Activities

The fluorogenic substrate dye-quenched (DQ)-gelatin was purchased from Thermofisher and was performed as described by the manufacturer and by Lima et al. [14] with the aim to test if the buffer extraction (BE) and purified protein (PP) exert inhibition of gelatinolytic activity by MMP-9 inhibition. In each experiment, positive (no protein fraction) and a negative (no enzyme) controls were performed for the samples, to correct possible proteolytic activities present in the samples under analysis. 

### 2.6. In Vitro Colon Cancer Cell Assays

#### 2.6.1. HT29 Cell Cultures

The human colon adenocarcinoma cell line, HT29 (ECACC 85061109), established from a 44-year-old Caucasian female, and obtained from ATCC was used during this work. HT29 cells were maintained as described by Lima et al. [14].

#### 2.6.2. Cell Migration Assay

For cell migration analysis, the wound healing assay was performed as described by Lima et al. [14] with some alterations. HT29 cells (4 × 10^5^ cells/well) were seeded in 24-well plates until reach 80% confluence. Cells were washed once with PBS to remove debris. Each well was completed with fresh medium containing 100 μg·mL^−1^ of the protein fractions under study. The migrated area after 48 h was calculated for each treatment and compared to the initial area at 0 h, to measure the area covered *de novo* by HT29 cells. This method allowed us to evaluate the inhibitory effect exerted by each protein sample on the cell migrating capacity.

#### 2.6.3. Cell Proliferation and Viability Assay

HT29 cultured cells were seeded in 96-well plates (2 × 10^4^ cells/well) and BE and PP samples were added to the growth medium at different concentrations: 5, 10, 50, and 100 μg·mL^−1^, and incubated for 48 h. The extracellular medium was collected, and the wells washed with phosphate buffer saline (PBS) to remove unattached cells. Cell proliferation and viability was determined using the standard 3-(4,5-dimethylthiazol-2-yl)-2,5-diphenyltetrazolium bromide (MTT) assay as described before [14].

### 2.7. Statistical Analysis

Statistical analysis was performed using SigmaPlot software (version 12.5) for samples comparison, using one-way analysis of variance (ANOVA). Tukey’s test was used to compare differences among treatments. All assays were performed in triplicate, in at least three independent times and the data are expressed as the mean ± standard deviation (SD) was used for comparing different treatments, using and two-way. 

## 3. Results and Discussion

Nutraceuticals and functional foods have become a huge trend today, reflecting a shift in the mindset of manufacturers, and consumers: rather than relying purely on medicinal products for the prevention and treatment of diseases, nutritional alternatives for both disease management and health promotion are readily available both in conventional foods and as individual supplements [1,23]. With the emerging nutraceutical industry, novel candidates have high applicability, as well as innovation and easiness to manufacture industrially fit perfectly within the goals of cost-effectiveness and innovation in industries [23].

### 3.1. MMPI Activity from L. albus Seeds Is Resistant to Heat Denaturation

**Figure 1:**
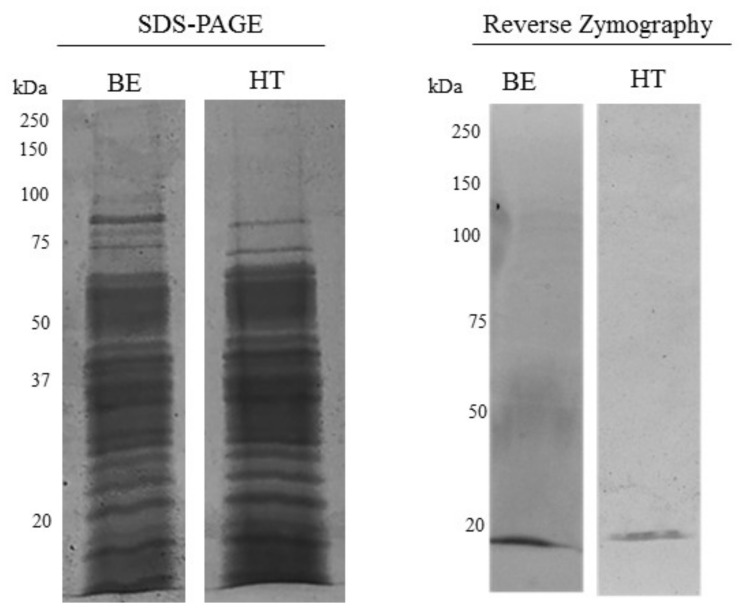
MMPI activity in *L. albus* resists heat treatment. The images of the polypeptide distribution between *Lupinus albus* seeds simply extracted with buffer (buffer extraction; BE) or after heat treatment (HT), and visualized by SDS-PAGE (**left**) or by reverse gelatin zymography (**right**). Each well was loaded with 50 µg.mL^−1^ of protein extracts in a 17.5% (*w*/*v* acrylamide) polyacrylamide gels. In the case of reverse zymography, the gels were copolymerized with gelatin and MMP-9.

Our previous findings showed that the MMP-9 inhibitory protein found in *L. albus* is an oligomer comprising fragments derived from two *Lupinus* seed storage proteins: δ-conglutin and, to a lower extent, β-conglutin [15]. Preliminary findings also showed that this oligomer could have a high stability to temperature [15]. This could be a very advantageous feature, as it could be used to eliminate a great part of the other proteins in *L. albus* seeds, which are easily denatured through heat. Under this context, we first set out to ascertain if the *Lupinus* seed MMPI found in Lima et al. [14] still presented MMPI activity after boiling. The polypeptide profile and the presence of MMPI bands by reverse zymography with MMP-9 included in the gel matrix were therefore evaluated. Figure 1 shows a representative image of the polypeptide profile of *L. albus* seed soluble protein extraction with tris buffer (buffer extraction; BE) and the same sample after heat treatment (HT) and centrifugation of the denatures proteins, visualized by SDS-PAGE (left) and by reverse gelatin zymography (right). 

SDS-PAGE profiles of the HT extracts show the presence of several polypeptide bands that survive heat denaturation at 100 °C, whilst the reverse zymography reveals the presence of only one major band, which maintains its biological activity after the heat treatment. The polypeptide band visible in both lanes of BE and HT presents a molecular mass lower than 20 kDa corresponding to the high MMPI activity present in deflamin as found earlier [14,16].

### 3.2. Sequential Extractions Allow the Isolation of Deflamin

Taking advantage of the fact that most of the oligomer in deflamin derived from δ-conglutin [16], that the MMPI band is heat resistant and that this MMPI fraction is highly soluble in water, a method for its isolation through sequential precipitations (appropriate for scaling-up to an industrial scale) was attempted. This method was developed based on previous protocols described for *Lupinus* conglutin’s isolation [18], whilst introducing some modifications, mostly targeted at the lower molecular mass of the heat resistant soluble proteins from *L. albus* seeds [14]. Hence, an initial water-soluble protein extract was obtained. This extract was boiled at 100 °C and centrifuged to eliminate part of the proteins, as represented in Figure 1. Subsequently, the remaining deflamin containing extract was concentrated by acid precipitation, which was demonstrated earlier to precipitate δ-conglutin [20]. The more hydrophilic fraction in the acid precipitate was then dissolved in 40% (*v*/*v*) ethanol, whilst the precipitated proteins were removed through centrifugation. The deflamin in solution was then precipitated overnight by adding ethanol up to 90% (*v*/*v*).

**Figure 2:**
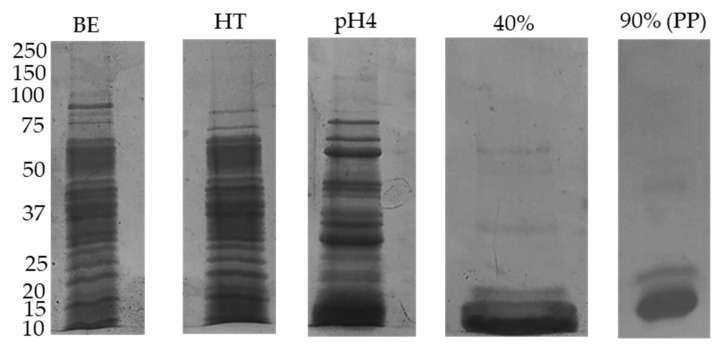
Representative images of the polypeptide profiles obtained after each step of the purification method as specified on the top of the gels. Each well was loaded with 25 µg of protein extracts in a 17.5% (*w*/*v* acrylamide) polyacrylamide slab gels. BE—Buffer Extraction; HT—Heat Treatment; pH4—Acid precipitation; 40%—40% *v*/*v* EtOH + 0.4M NaCl; 90% (Purified protein—PP)—deflamin obtained by 90% (*v*/*v*) EtOH.

The representative images of the electrophoretic soluble polypeptide profiles obtained after the several sequential extractions are shown in Figure 2. 

Analysis of the polypeptide profiles following each step of the isolation method depicted in Figure 2 revealed the gradual purification of a polypeptide fraction with a molecular mass below 20 kDa, which was termed deflamin and which matched the band obtained in reverse zymography (Figure 1 right). 

The combined use of heat denaturation, acid precipitation, and ethanol solubilization assures the removal of possible contaminants such as saponins, phenolic compounds, and other known secondary metabolites present in legume seeds [24]. The final deflamin (purified protein) obtained by 90% (*v*/*v*) ethanol solubilization yields the lower molecular mass, more hydrophilic, polypeptide fraction.

In order to determine if the isolate was deflamin, we determined its sequence through mass spectrometry. Mass sequencing of the polypeptide fraction (PP) revealed the presence of the fragments of the two of these major proteins, β- and δ-conglutin present in deflamin [16], as described in Table 1, hence corroborating that the isolate obtained through our sequential method was indeed deflamin. 

### 3.3. Deflamin Isolate Maintains its Activity

To determine if the deflamin obtained through our sequential method still maintained its MMPI activity, we compared the activities against MMP-9 and colon cancer cell migration in both the buffer extraction (BE) and the purified protein (PP). 

**Figure 3:**
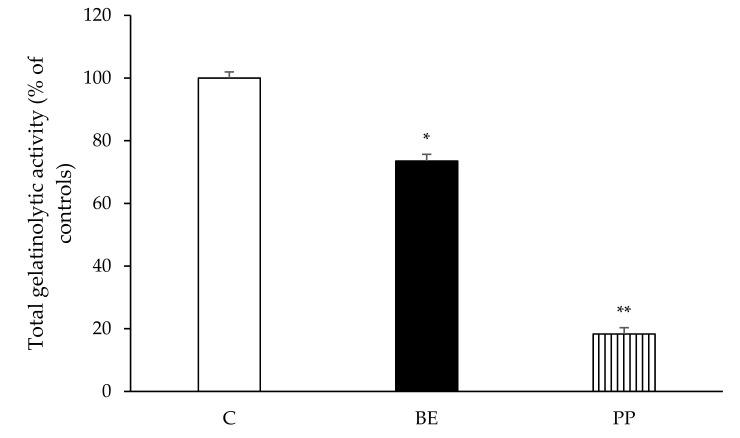
The effect of buffer extraction (BE) and purified protein (PP–deflamin) on the proteolytic activity of MMP-9. Each well was loaded with 50 µg.mL^−1^ of protein and gelatinolytic activity was measured by the DQ-gelatin assay. MMP-9 activity is expressed as relative fluorescence as a % of controls and correspond to the means of at least three replicate assays (n = 3) ± SD. * *p* < 0.05, ** *p* < 0.001.

Results are shown in Figure 3. Buffer extraction (BE) and deflamin (PP) protein fractions were used to assess their inhibitory activity upon the proteolytic activity of MMP-9 on DQ-gelatin. The positive control (C) inhibits MMP-9, resulting in 100% proteolytic activity for this protease.

At the protein concentration tested (50 µg·mL^−1^), Figure 3 shows that all samples were able to significantly inhibit MMP-9 proteolytic activity. However, significant differences (*p* < 0.05) were observed among the samples analyzed, with the highest inhibition level detected for PP, which induced a very significant (*p* < 0.001) reduction of MMP-9 activity greater than 80%. Indeed, as deflamin is purified, its apparent inhibitory effect as an MMPI increases and its effect is significantly higher than BE (*p* < 0.05). This suggests that although *L. albus* total protein pool contains deflamin and can inhibit MMP-9 activities as described by Lima et al. [14], isolated deflamin is most likely more effective that the consumption of lupin as a functional food. This is further corroborated by the fact that delta conglutin was assessed to be around 3 to 4% of the seed weight [25]. 

Subsequently, we further tested our PP for its ability to reduce cancer cell migration in HT29 cells, while comparing it to the total extract of *L. albus*, using the same inhibitory concentrations found earlier [14,15]. 

**Figure 4:**
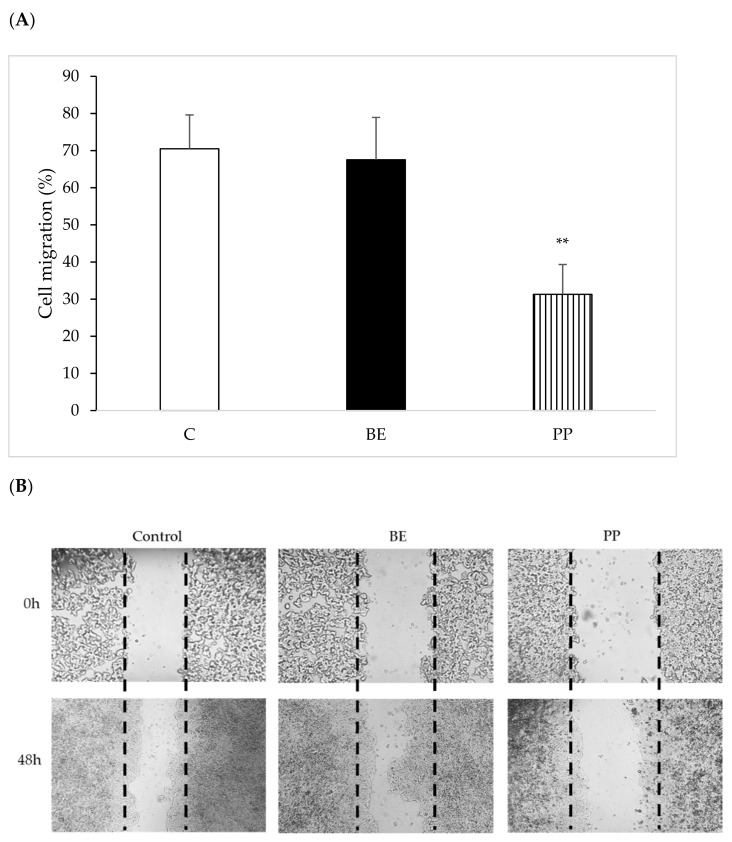
HT29 cell migration after exposure to 100 μg·mL^−^^1^ of the protein fractions under study: buffer extraction (BE) and deflamin (PP), as determined by the wound healing assay. (**A**) Relative migration rates. Values are the averages of at least three assays ± SD, and are expressed as a % the wound migration in relation to 0 h. (**B**) Representative pictures of cell migration demonstrating the inhibitory effect of deflamin on HT29 cell migration. ** *p* < 0.001.

Figure 4 indicates the effect of each protein fraction on cancer cell migration after 48 h of exposure to the total extract (BE) and purified protein (PP-deflamin).

Results show that isolated deflamin presented the highest inhibition in migration rates when compared to the other deflamin-containing protein samples studied (*p* < 0.001), inducing a 60% reduction in cell migration rates. Furthermore, at the concentration used, deflamin was also statistically different from controls (*p* < 0.05) whilst the BE sample remained statistically similar to controls (*p* > 0.05), once again corroborating the higher efficiency of the isolated deflamin in the PP fraction, already noted in Figure 3.

This means that, as the purification methodology proceeds from the initial total protein extract to isolated deflamin, its biological activity gradually increases, reaching a maximum with isolated deflamin. This result was expected given the concomitant increment in deflamin specific activity as the other proteins are gradually removed along the purification process. Indeed, as comparative tests performed after each purification step use identical amounts of proteins from each sample, as the degree of deflamin purification increases, the amount of deflamin relative to total protein in each fraction also increases, justifying the increment in deflamin bioactivity when one moves from less pure to purer deflamin fractions.

**Figure 5:**
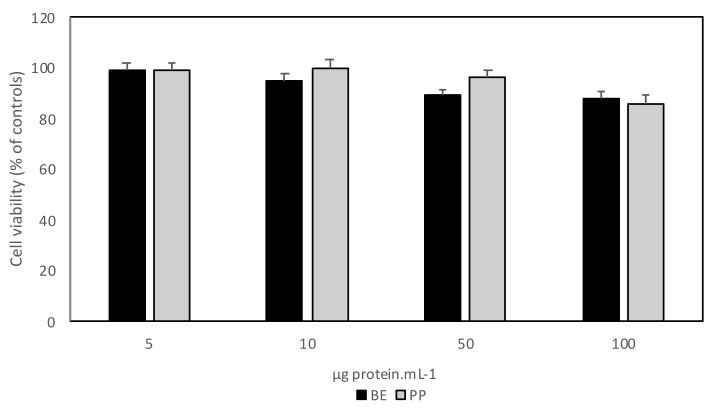
HT29 cell viability after a 48 h exposure to different concentrations of BE and PP fractions. Cells were grown for 48 h in the presence of 100, 50, 10, and 5 µg protein·mL^−^^1^ and stained with MTT. Values represented are the averages of three replicate experiments (n = 3) ± SD and are expressed as a percentage of the control.

The PP inhibitory activity is similar to previously reported inhibition of the albumin fractions from L. albus, Cicer arietinum and Glycine max, with 68, 63, and 61% reductions, respectively [14]. It also reports the use of different plant extracts with MMP-9 inhibitory activities from two species of *Aloe* [26] and demonstrated similar results and a known MMP-9 inhibitor, doxycycline in the same conditions, induced similar reductions in cell migration [6]. However, in these same reports, and under these conditions, doxycycline yielded a cell viability of 50%, whereas the Aloe species yielded a cell viability of 45 and 35%. Our previous results had demonstrated that, although they impaired cancer cell growth migration, the albumin lupin extract (BE) and isolated deflamin present no cytotoxicity to HT29 cells and other cell lines [14,15,27]. Nonetheless, it was deemed important to ascertain that our PP extract presented no effect on cell viability. We therefore set out to evaluate the effect of different concentrations of BE and PP on HT29 cell metabolism and proliferation, using the standard MTT method (Figure 5).

Results show that at the studied concentrations, cell viability was not significantly reduced, which agrees with our previous results [15,27], ascertaining the PP extract’s safety for food purposes.

### 3.4. Deflamin Is the First Proteinaceous MMPI That Can Be Purified by a Cost-Effective and Up-Scalable Procedure 

There is abundant evidence in the published literature concerning the MMPI activities of many edible foodstuffs. For example, Cyr [28] provides a huge list of plants (either stressed or non-stressed) whose aqueous, ethanolic, or organic extracts exhibit inhibitory activity upon human MMP-2 and MMP-9 enzymes. Legume seeds in particular have been long recognized by containing a variety of proteinaceous enzyme inhibitors, such as BBIs [29]. 

Nonetheless, although the presence of MMPIs of natural occurrence may be considered frequent in plant tissues, virtually all these secondary metabolites suffer from the same disadvantages of synthetic MMPIs [30]. Even if we consider plant-derived proteins with MMPI activity, they suffer from at least one of the following limitations, handicaps, disadvantages, or weaknesses when we consider their possibility of clinical and/or nutraceutical application: toxicity; chemical inactivation (e.g., denaturation), or destruction (e.g., proteolysis) during the digestive process; absorption into the blood stream of the whole protein or a part of it, with or without triggering immunogenic (i.e., IgG) or allergenic (i.e., IgE) responses; destruction and/or denaturation during boiling (e.g., during cooking); low water solubility, no specificity towards the gelatinases, high dose requirements, lack of a specific and usually high-cost and inefficient methods of isolation, which prevent MMPIs in general to undergo efficient scaling-up to an industrial level [30,31,32,33,34]. 

These certainly explain, for the most part, why there is not yet a single plant-derived biological compound that found successful application in human health and nutrition at the level of MMP inhibition. 

Our isolated deflamin protein precipitate reported in this work surpasses all of these constraints, as it is resistant to boiling and is an enzyme inhibitor; on the other hand, the sequential precipitation method developed is simple, cost-effective, and easily applied in an industrial context. As an oligomer of polypeptides that occurs naturally in lupin seeds, it does not pose the problem of toxicity in higher doses [15] that most phenolic compounds and other bioactive secondary metabolites do, and the use of the acid and ethanol precipitations assures the removal of possible toxic contaminants as well as higher molecular mass proteins. 

The yield of the extraction procedure was found to be 0.1% per 100 g of dry seed and 0.5% per total protein content of the seed, which corresponds to 100 mg and 520 µg, respectively. These results corroborate that deflamin is indeed present in very low concentrations in the seed, hence the lower activities observed in the BE fractions. It also suggests that the consumption of lupin alone may not provide enough deflamin to induce the same effects that its isolated form can provide. It is important to notice that the low yields of the extraction procedure are not due to the method itself, but rather to the low amount of deflamin in the seed. Still, the relative ease of the procedure and the possibility to up-scale to larger amounts, in a cost-effective and simple manner, using filtrations and flow centrifugation as well as low-cost reagents such as ethanol suggest a high potential for industrial production.

In our previous works we determined that the required dose of isolated deflamin for physiological activity in induced colitis models in vivo would be 15 mg.kg^−^^1^. Since our PP extract presents considerable purity in terms of other proteins and the sequential precipitations assure the removal of other possible contaminants, such as water-soluble phenolics and sugars, we assume that the same concentration of the PP. Indeed, our preliminary in vivo results have corroborated this (data not shown). Overall, the method provided here seems to present an effective way to deliver this nutraceutical in functional foods, without much cost or effort. Usually, bioactive proteins such as deflamin are purified using costly and time-consuming methods such as chromatographic separations and sometimes recombinant protein production. This requires costly reagents, incompatible to human nutrition and complex equipment. In fact, one of the main challenges in bioactive compounds use in functional diets is exactly their isolation and delivery. Therefore, the use of simple sequential precipitations can be rather advantageous to use this nutraceutical as a food ingredient in preventive diets. 

The present method can be applicable to both the food industry and the clinic. Furthermore, since the predicted MMPI activities were also found in other legume seeds such as chickpea and soy [14], we expect that this same procedure can be used to yield efficient MMPIs from other species as well. Hence, from an ecological and agronomical point of view, the use of Lupinus and other pulses as sources of nutraceutical MMPIs using this method can stimulate the selection of more locally sourced food ingredients, so the proposed strategy will contribute to sustainability as well.

## 4. Conclusions

Here, we report a simple procedure aimed at fractionating the main proteins of lupin seeds, whilst using the known unique features of deflamin, to isolate this potent MMPI from *L. albus* seeds. This is, to the best of our knowledge, the first effective method for isolating a proteinaceous MMPI from food sources that is scalable to an industrial level, in a cost-effective manner. Furthermore, the fact that isolated deflamin is efficient in inhibiting MMP-9 and reducing cancer cell migration suggests its high potential for a vast array of clinical uses. Since MMP-9 is closely involved in inflammation [13,35] as well as in oncologic processes [2,13], the MMPI deflamin could possibly be used in both approaches treatments, especially those related to the digestive tract, such as colorectal cancer and inflammable bowel diseases.

## 5. Patents

DEFLAMIN: Therapeutic protein. 

PCT International Patent Application No. PCT/EP2017/075020. Filed on 30 September 2017. Available at https://patentscope.wipo.int/search/en/detail.jsf?docId=WO2018060528&recNum=4&office=&queryString=FP%3A%28075020%29&prevFilter=&sortOption=Pub+Date+Desc&maxRec=48. (accessed on 20 May 2021).

## Figures and Tables

**Table 1:** Mass spectrometry analyses of *L. albus* deflamin (PP).

N	Unused	Total	%Cov(95)	Accession	Name	Species	Peptides (95%)
3	12.48	12.48	36.25	P09931 CGD2L_LUPAN	Conglutin delta-2 large chain	LUPAN	8
98	2.13	2.22	8.29	Q53HY0 CONB1_LUPAL	Conglutin beta 1	LUPAL	3
